# The role of extracellular vesicles in animal reproduction and diseases

**DOI:** 10.1186/s40104-022-00715-1

**Published:** 2022-06-10

**Authors:** Sangiliyandi Gurunathan, Min-Hee Kang, Hyuk Song, Nam Hyung Kim, Jin-Hoi Kim

**Affiliations:** 1 Department of Stem Cell and Regenerative Biotechnology, Konkuk University, Seoul, 05029 Korea; 2 Guangdong Provincial Key Laboratory of Large Animal models for Biomedicine, Wuyi University, Jiangmen, 529020 China

**Keywords:** Embryos, Extracellular vesicles, Implantation, MicroRNA, Pregnancy disorders

## Abstract

Extracellular vesicles (EVs) are nanosized membrane-enclosed compartments that serve as messengers in cell-to-cell communication, both in normal physiology and in pathological conditions. EVs can transfer functional proteins and genetic information to alter the phenotype and function of recipient cells, which undergo different changes that positively affect their structural and functional integrity. Biological fluids are enriched with several subpopulations of EVs, including exosomes, microvesicles (MVs), and apoptotic bodies carrying several cargoes, such as lipids, proteins, and nucleic acids. EVs associated with the reproductive system are actively involved in the regulation of different physiological events, including gamete maturation, fertilization, and embryo and fetal development. EVs can influence follicle development, oocyte maturation, embryo production, and endometrial-conceptus communication. EVs loaded with cargoes are used to diagnose various diseases, including pregnancy disorders; however, these are dependent on the type of cell of origin and pathological characteristics. EV-derived microRNAs (miRNAs) and proteins in the placenta regulate inflammatory responses and trophoblast invasion through intercellular delivery in the placental microenvironment. This review presents evidence regarding the types of extracellular vesicles, and general aspects of isolation, purification, and characterization of EVs, particularly from various types of embryos. Further, we discuss EVs as mediators and messengers in reproductive biology, the effects of EVs on placentation and pregnancy disorders, the role of EVs in animal reproduction, in the male reproductive system, and mother and embryo cross-communication. In addition, we emphasize the role of microRNAs in embryo implantation and the role of EVs in reproductive and therapeutic medicine. Finally, we discuss the future perspectives of EVs in reproductive biology.

## Introduction

**Fig. 1:**
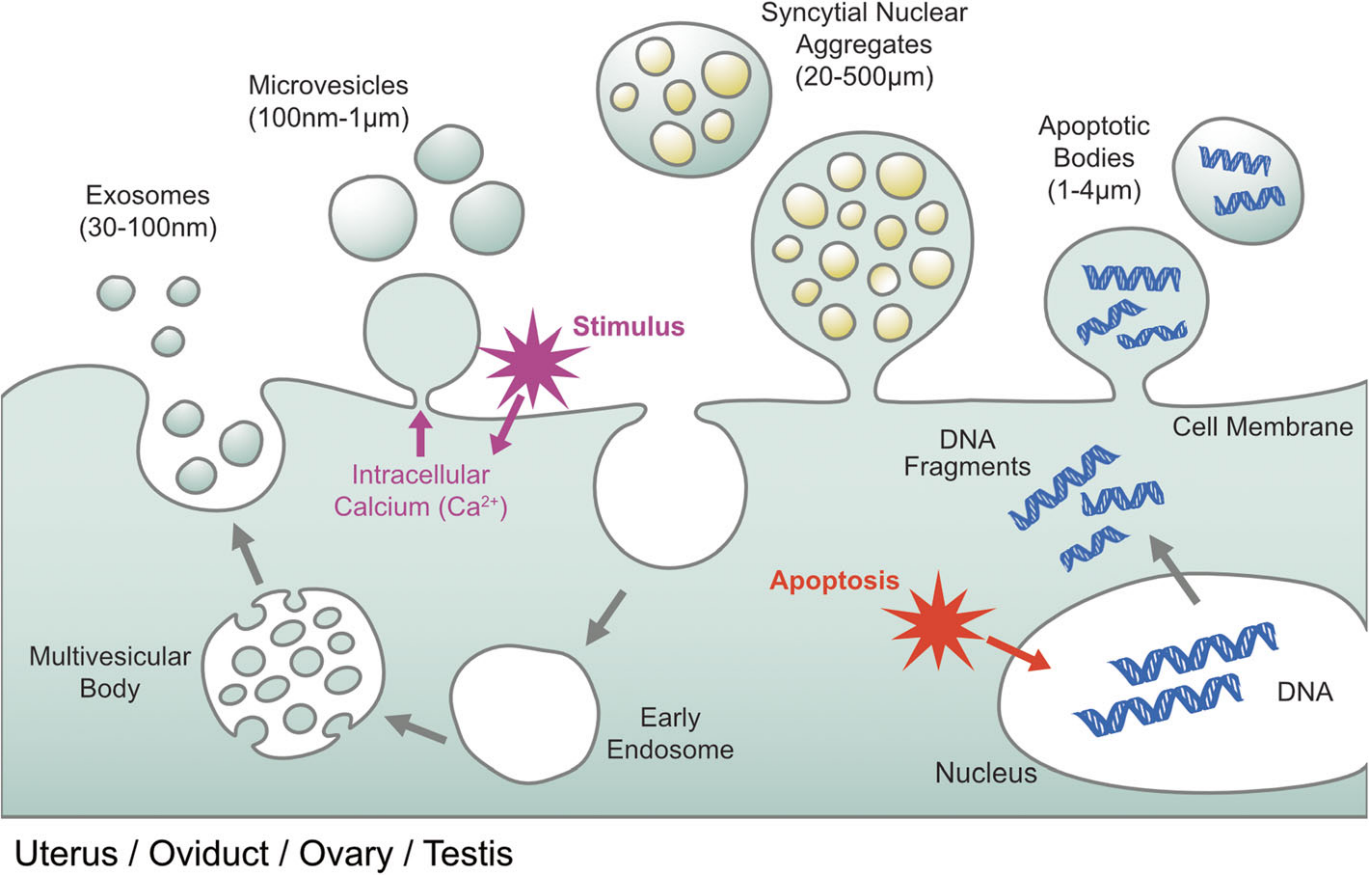
Biogenesis of extracellular vesicles in male and female reproductive systems. EVs are composed of functional proteins, mRNA, and microRNA. In particular, the protein content of EVs depends on the cell type from which they are secreted. Biogenesis of extracellular vesicle (EV) subtypes such as exosomes, MV, syncytial nuclear aggregates and apoptotic bodies. EVs are intraluminal vesicles which are released when a multivesicular body fuses with the cell membrane through exocytosis. MVs are formed by outward shedding of the cell membrane into extracellular space. Apoptotic bodies are generated when cells undergo apoptosis. The macromolecular components of EVs may play a significant role in cellular functions and pathological states during ovarian and uterus cycling, implantation of female as well as male reproduction

Extracellular vesicles (EVs) are heterogeneous and nanosized membranous vesicles secreted by a wide range of cells throughout the body. They are found in various body fluids, such as blood, urine, saliva, and breast milk. EVs are known for their ability to carry significant phenotype-altering cargo, such as transcription factors and microRNAs [1]. Based on their biogenesis and size, EVs are classified as exosomes (50~150 nm), microvesicles (100~1000 nm), or apoptotic bodies (500~4000 nm) [2, 3] (Fig. 1). Generally, EVs play a significant role in cellular dumping or the release of waste materials. EVs deliver various cargoes, including mRNAs, microRNAs (miRNAs), lipids, proteins, and nucleic acids, for long-distance communication between cells [3, 4]. Pathological cells, such as cancer cells, secrete specific EVs with different compositions that can be used as diagnostic markers for certain diseases and can also be used for monitoring disease progression [5–7]. Extracellular vesicle secretion has been observed in various reproductive cells, such as follicular cells [8], oviductal cells [9], embryos produced in vitro [10], and endometrial cells [11]. EVs regulate various reproductive physiological functions, including ovarian follicle development, oocyte maturation and fertilization, early embryo development, and endometrial–conceptus crosstalk [8, 9, 12–14]. Exosomes are derived from the inward pushing of the plasma membrane, which is typically 30–150 nm [15]. The endolysosomal system comprises a complicated and dynamic membranous network that begins from the early to late sorting of endosomes, formation of multivesicular bodies (MVBs), and fusion with the plasma membrane for secretion [3]. MVB formation is carried out by machinery that may either be endosomal sorting complexes required for transport (ESCRT)-dependent or ESCRT-independent. Exosomes and MVs are produced and secreted during normal cellular activity; in contrast, apoptotic bodies are larger in size (500–4000 nm), contain cell organelles within them, and are released during apoptosis, which is one of the major mechanisms of cellular death [16]. Studies have reported that EVs from bovine follicular fluid from small follicles (3–5 mm in diameter) and large follicles (>9 mm in diameter) induce cumulus expansion during in vitro maturation [13].

Due to their unique composition, cells of origin, and pathological characteristics, EVs are used to diagnose various diseases. EVs contain microRNAs (miRNAs) and proteins, which regulate inflammatory responses and trophoblast invasion through intercellular delivery in the placental microenvironment [17]. Maternal circulating EVs play a significant role in the formation of pro-inflammatory environments and endothelial cell dysfunction in the placenta [18]. EVs secreted from the embryo are involved in both the dialogue with the maternal endometrium [19], and in self-paracrine regulation [20]. Pig and human models show that EVs can be secreted from the trophectoderm and stimulate the proliferation of endothelial cells in vitro, thus becoming potential regulators of maternal endometrial angiogenesis [19, 21]. Embryo implantation is a crucial step in pregnancy, and failure of embryo implantation is a major limiting factor in early pregnancy and assisted reproduction. Implantation governs various physiological parameters, including embryo viability, endometrial receptivity, and embryo-maternal interactions. Embryo implantation is regulated by various types of biomolecules, particularly microRNAs , which function as transcriptional regulators of gene expression. miRNAs not only act in the cells, but can also be released by cells into the extracellular environment through multiple packaging forms, facilitating intercellular communication and providing indicative information associated with physiological and pathological conditions [22].

The function of EVs in human reproduction depends on the load of EVs and their ability to interact with receptor cells to deliver various types of cargo. EVs specifically bind to target cells, depending on the EV content and the specific receptors of the target cells or tissues. Because of their stability, versatility, and their ability to target recipient cells with specificity and transfer genetic and protein material through biological barriers [23], EVs are considered novel diagnostic and therapeutic tools in reproductive biology. Their therapeutic potential depends on their cargo composition. The release of EVs from the placenta is regulated by a number of factors that arise from the placenta. Changes in EV content and functions might be used as diagnostic biomarkers in female fertility studies [24]. This review discusses the different types of EVs, general aspects of isolation, purification, and characterization of EVs, particularly from various types of embryos. Further, we discuss EVs as mediators and messengers in reproductive biology, the effects of EVs on placentation and pregnancy disorders, the role of EVs in animal reproduction, male reproductive system, and mother and embryo cross-communication. In addition, we emphasize the role of microRNAs in embryo implantation and the role of EVs in reproductive and therapeutic medicine. Finally, we discuss the future perspectives of EVs in reproductive biology.

## Types of EVs

### Exosomes

EVs are classified as exosomes, microvesicles, and apoptotic bodies, based on various parameters, such as cellular origin, biophysical and biochemical characteristics, biological function, and biogenetic pathway. Exosomes are nano-sized particles that are trafficked through the endosomal pathway. Endosomal sorting complexes required for transport (ESCRTs) are important for the biogenesis of multivesicular bodies. Exosomes are derived from the inward budding of the limiting membrane of late endosomes, facilitating the formation of intraluminal vesicles (ILVs). Exosome formation is governed by two different mechanisms, ESCRT-dependent and independent; mechanisms including neutral sphingomyelinase/ceramide formation and involvement of ARF6/PLD2 have also been reported to occur [25, 26]. ILVs released from MVBs into the plasma membrane are called exosomes. On the other hand, the fusion of ILVs with lysosomes is mainly for the degradation of their contents [27]. Exosome secretion is regulated by various factors, such as members of the Rab guanosine triphosphatase (GTPase) RAB27A, RAB27B, RAB11, and RAB35. The machinery involved in the biogenesis of MVB and exosomes varies between tissues and cell types, which is governed by specific metabolic needs [23, 28]. Almost all secreted exosomes are between 30 nm and 150 nm; in some cases, it can be up to 200 nm, which is similar to the size of viruses [28–30]. Exosomes are typically characterized by the expression of surface markers, such as CD9, CD63, CD81, Alix, TSG101, and flotillin, as well as other markers [31]. Exosomes have been reported in various cells and parts of the body including within the zona pellucida [32, 33]. The human blastocyst cavity contains exosomes that are CD63^+^ and CD81^+^ [34]. Previous studies have reported that small EVs are located in various reproductive cells, including follicular fluid [8], oviductal fluid [9], secreted by embryos in culture media [35–37] and in endometrium flushing [11]. EVs from oviductal fluid facilitate oocyte and embryo quality [14]. Preimplantation embryos secrete exosomes from CD9^+^ cells through exocytosis or endocytosis [38, 39].

### Microvesicles

MVs are a population of EVs that are formed and released directly from the cell plasma membrane by outward budding and fission from viable cells [40, 41] and are regulated by multiple mechanistic approaches. MVs are derived from budding events nucleated by the protein ARRDC1, which is recruited to the plasma membrane along with elements of the ESCRT pathway, generating 50 nm vesicles [42]. Another protein, Bin-1 (ampiphysin), facilitates the formation of curvature when recruited to the membrane. The formation and release of MVs are triggered by the remodeling of membrane proteins and lipid redistribution, which modulate membrane rigidity and curvature [43, 44]. ARF6 is a guanosine triphosphate–binding protein, a marker of MVs, and is implicated in the regulation of cargo sorting and promotion of the budding and release of MVs through the activation of the phospholipase D metabolic pathway [44, 45]. MVs play significant roles and various functions, including cancer cell invasiveness, transformation potential, disease progression and drug resistance, regulation of autoimmune diseases, immune system modulation and coagulation, embryo–maternal crosstalk, and embryo self-regulation [46–49].

### Apoptotic bodies

Apoptotic bodies are produced as a result of cell death, alteration of several morphological changes, including membrane blebbing, membrane protrusion formation [50]. Apoptotic cell-derived extracellular vesicles, otherwise called ABs, are a group of subcellular membrane-bound extracellular vesicles generated during the decomposition of dying cells. ABs can be generated by many types of cells, such as stem cells, immunocytes, precursor cells, osteoblasts, and endothelial cells [51]. The production of ABs occurs in a dose-and time-dependent manner and is regulated by various factors, such as Rho-associated protein kinase (ROCK1) [52–54] and myosin-light chain kinase (MLCK) [55]. Inhibition of ROCK1, MLCK, and Caspases prevents the production of ABs. ABs are produced by nuclear shrinkage and plasma membrane blebbing in cells undergoing programmed cell death and MLCK contributes to the packaging of nuclear material into ABs [56]. Actomysin also plays a role in AB production by increasing cell contraction, hydrostatic pressure, and the formation of blebs [57]. The membrane of ABs reflects the main changes occurring on the cell surface of apoptotic cells. Apoptotic microvesicles ranging from 0.1 to 1 μm in diameter and small exosome-like EVs are released during apoptotic conditions [58, 59]. ABs are formed by the fragmentation and packaging of cellular organelles, such as the nucleus, endoplasmic reticulum (ER), or Golgi apparatus into these vesicles [16, 60], which range from 1 to 5 μm. The ER membrane is fragmented and forms vesicles smaller than ABs that sediment at higher centrifugal forces. ABs are divided into two types based on the type of cargo: DNA-carrying ABs, and cytoplasm-carrying ABs [61]. ABs are typically characterized by cytoskeletal and membrane alterations, including the translocation of phosphatidylserine (PS) from the inner to the outer leaflet of the lipid bilayer [62]. VDAC1 is an apoptotic marker that forms ionic channels in the mitochondrial membrane and plays a role in triggering apoptosis; it is specifically localized in the vesicular fraction [63]. Another AB marker is calreticulin, an ER protein which is located in the subcellular localization. ABs are associated with the immune system [64–66]; they express chemokines and adhesion molecules, such as CX3CL1/fractalkine and ICAM3, and MHC class II molecules that can facilitate antigen presentation to CD4^+^ T cells and activation of immunological memory [67]. ABs are being developed as an essential tool in cell-to-cell communication between damaged and healthy cells. ABs may stimulate the proliferation of resident stem/progenitor cells, improve tissue regeneration, and replace damaged cells [68, 69]. ABs originating from different cell types have been shown to promote various functions. In the hepatic stellate, ABs can promote differentiation and cell survival [70]. ABs containing DNA from endothelial cells induce the proliferation and differentiation of human endothelial progenitor cells in vitro [71]. ABs containing microRNAs of cardiomyocytes enhance the proliferation and differentiation of resident SCs in vitro [72]. Administration of ABs carrying miR-126 inhibits atherosclerosis and induces CXCL12-dependent vascular protection [73]. ABs from cardiomyocytes enhance the proliferation and differentiation of resident stem cells (SCs) by transporting specific miRNAs [72], while ABs containing miR-221 and miR-222 derived from macrophages promote the proliferation of epithelial cells [74].

## Isolation, purification and characterization of EVs

### Isolation and purification of EVs

Isolation, purification, and characterization of EVs are essential for the application of EVs in a variety of fields. In particular, homogeneous separation and high yield are important in clinical applications. In this study, we provide a brief account of various isolation and purification methods. Differential centrifugation is the most commonly used method for the isolation of EVs [75]. The first step is low speed centrifugation at 300 × *g* for 10 min, which is required to eliminate cells. The second centrifugation at 2000 × *g* is for pelleting membrane debris and dead cells. The third centrifugation at 10,000 to 20,000 × *g* for 30 min is performed to pellet microvesicles. After these three steps, supernatants are collected and a fourth centrifugation step is carried out at 100,000 to 200,000 × *g* for 70 min to isolate exosomes. In this step, pellets are collected and washed with phosphate-buffered saline (PBS), and centrifuged again under the same conditions to remove impurities. Although the centrifugation process provides EVs, ultracentrifugation cannot remove contaminating lipoproteins from biological samples, such as blood. Hence, gradient centrifugation and/or other chromatography techniques are essential to remove impurities [76–79]. To improve the population purity of EVs, gradient step centrifugation is indispensable. In this step, the pellet is resuspended in PBS, loaded into a sucrose cushion or gradient, and ultracentrifugation is carried out. The vesicles are recovered either from the bottom of the tube or from a specific fraction of the gradient, depending on their buoyant density [75, 76, 80–83]. Ultracentrifugation is used to improve purification of EVs. Otherwise, ultrafiltration is utilized, where filtration membranes of different molecular mass cutoffs are centrifuged at moderate centrifugal forces. This simple and rapid method allows the concentration of vesicles at the interface of the filters. However, the filtration method has some disadvantages, such as a decreased yield, and the use of pressure can cause the EVs to deform or break into smaller vesicles.

Size-based exclusion chromatography is an efficient chromatography technique that separates particles based on their size, which can be used to separate and purify EVs from proteins in complex biological samples. However, when used to purify EVs from plasma or serum, this technique cannot efficiently separate EVs from lipoproteins of similar size [84]. To purify EVs from lipoprotein, density gradient ultracentrifugation followed by size exclusion chromatography is needed [79]. Other types of chromatography may also be used to purify EVs. Affinity purification and ion exchange are used for the purification of EVs from biological samples [85]. Immuno-affinity columns selectively purify EVs using capture agents, including heparin, tetraspanins, and epithelial cell adhesion molecule (EpCAM) [86–88]. Anion exchange chromatography is a simple, efficient, scalable, and dependable method for the isolation of EVs from cell culture supernatants [89, 90]. Negatively charged EVs bind to positively charged columns, and EVs are eluted from the column using increasing concentrations of salt. The precipitation method is a rapid, feasible, and cost-effective method that allows EVs to be pelleted by low-speed centrifugation using polyethylene glycol [91] or Exoquick [92]. However, the purity of EVs from the precipitation method is not absolute; it may contain EVs with other proteins and lipoproteins. In addition, EVs purified from precipitation affect the viability and biological activity of recipient cells and EVs [93, 94]. Recently, isolation of EVs has attracted microfluidic chip technology, which is useful for the capture and analysis of EVs from small volumes of clinical samples and shows promise for liquid biopsy diagnosis of disease [95]. Microfluidic devices have been engineered for immuno-capture using tumor-specific antigens, such as human epidermal growth factor receptor (HER2) and prostate-specific antigen (PSA) [96].

### Characterization of EVs

The characterization of EVs is an essential step in clinical applications. The first and leading technique is microscopy, which is used for morphology and size analysis. In particular, electron microscopy techniques are the only method available to visualize the appearance of EVs, which are generally cup-shaped [97]. Atomic force microscopy (AFM) is an alternative method for analyzing the size distribution and quantity of EVs within a sample. The use of aqueous media is advantageous because it permits the maintenance of the physiological properties and structure of EVs [98, 99]. The combination of AFM and microfluidic techniques allows for the consecutive isolation and characterization of EVs.

The size distribution of EVs can be measured by nanoparticle tracking analysis (NTA), a light scattering technique which is now widely used for the assessment of EV size distributions and concentrations in the range of 50 to 1000 nm [100]. This technique is based on the inherent Brownian motion of particles in a solution. Dynamic light scattering (DLS), which uses the same principle, can be also used to assess the EV size distribution. A tunable resistive pulse is a novel and less expensive technique for the analysis of particle size distributions within the range of 30 nm to 10 μm. The system is composed of a thermoplastic polyurethane membrane containing nanopores that are selected based on size requirements. On the other hand, flow cytometry is used to measure the size distribution, concentration, and qualitative characteristics of EVs within a sample. Light scatter flow cytometry can measure within the range of 300 nm to 500 nm; however, exosomes cannot be measured because the size of exosomes is between 30 nm and 150 nm. Innovations in flow cytometry uses fluorescent labeling of EVs, which reduces the lower limit of detection to ~100 nm. Finally, antibodies coupled with surface markers of EVs can be used to measure nanosized EVs [101–104].

### Isolation, purification and identification of EVs from reproductive cells/embryos

EVs play a significant role in the male and female reproductive tracts, making connections between the reproductive tract and immature germ cells, or between the mother and the developing embryo. As such, the uses of EVs have potential implications for the establishment of a successful pregnancy or understanding associated pathological conditions [105]. Hence, we focused specifically on the isolation, purification, and identification of exosomes from embryos.

The isolation and purification of exosomes from somatic cell-cloned embryos were described previously [76, 106, 107]. Embryos were cultured for 3 d on defined medium, and then the medium was subjected to differential centrifugation to remove various debris at 4 °C (300 × *g*, 10 min to remove cells; 2000 × *g*, 10 min to remove dead cells; and 10,000 × *g* for 30 min to remove cell debris, macroparticles, and apoptotic bodies). The supernatants were then ultracentrifuged at 100,000 × *g* for 70 min in 14 mm × 95 mm ultra-clear centrifuge tubes (Beckman). The pellets from a single sample were pooled, resuspended in PBS, and centrifuged again at 100,000 × *g* for 70 min. Each pellet was resuspended in 30 μL of the defined medium to supplement the renewed culture medium. Exosomes were identified as previously described [106].

The isolation of EVs from bovine embryos was described previously [108]. Bovine embryos were cultured on conditioned media and then sequential centrifugation was carried out to remove larger particles, which were then filtered using 0.2 μm syringe filters and used for sample dilution and EV isolation. The culture medium was then subjected to double centrifugation. Initially, the diluted samples were centrifuged at 400 × *g* for 10 min at 4 °C to remove dead cells and debris, and the collected supernatants were further centrifuged at 2000 × *g* for 10 min to remove apoptotic bodies. Finally, EV isolation was performed using qEVsingle size exclusion columns. Fractions were collected and pooled as EVs were eluted in these fractions. The size and concentration of EVs in the pooled fractions were determined using a nanoparticle tracking analyzer.

Burkova et al. [109] reported on the methods for isolation, purification, and identification of exosomes from the placenta. The human placenta is a highly specialized organ that connects mother and fetus organisms, and it protects, nourishes, and regulates the growth of the embryo. Placenta extract preparations were obtained from total placentas. Exosomes were isolated from the placenta using various methods. Supernatants were subjected to sequential centrifugation twice at 10,000 × *g* for 40 min at 4 °C and once for 16,500 × *g* for 20 min, and the supernatant was filtered through a 0.22-μm filter. The filtered supernatant was ultracentrifuged at 100,000 × *g* for 2 h. After the first centrifugation, the pellet was resuspended in 8 mL of TBS. The resuspended pellet was ultracentrifuged twice at 100,000 × *g* for 2 h. The precipitate was resuspended, filtered through a 0.1-μm filter, and purified further using gel filtration on Sepharose 4B columns. Exosomes derived from placentas underwent various purification steps. Transmission electron microscopy revealed the aggregation of exosomes, microparticles, and amorphous protein [109]. The isolated exosomes contained the typical surface markers CD81, CD63, and tetraspanins.

EVs have been isolated and characterized from human blastocoel fluid (BF), as described previously [34]. BF samples were collected from human embryos on the fifth day of development from patients undergoing IVF cycles. Exosomes were isolated from BF, and morphological and molecular characterizations were performed using various analytical techniques, such as scanning electron microscopy (SEM) and nanoparticle tracking analysis (NTA). SEM observation revealed vesicles of spherical shape with an average diameter of 75 ± 3 nm and full width at half maximum (FWHM) of 38 ± 8 nm, compatible with exosome size [34].

Simon et al. [33] reported the isolation, identification, and characterization of EVs from mouse embryos. Embryos from 10 animals were used for the identification and phenotypic characterization of EVs using electron microscopy and immunogold. Embryos were collected from conditioned media at day E4.5 and centrifuged at low speed (300 × *g*, 10 min) to remove larger debris. The resulting supernatant was centrifuged at 2000 × *g* for 10 min to recover apoptotic bodies, as previously described [110]. It was subsequently ultracentrifuged at 185,000 × *g* for 70 min in a P50A3 Hitachi rotor (Hitachi, Tokyo, Japan) to collect non-apoptotic EVs (naEVs) that included MVs and exosomes in the same fraction. TEM images revealed the presence of MVBs in the cytoplasm of murine oocytes. The presence of MVBs was also observed in the blastomeres at different embryonic developmental stages (E2.5 and E3.5), migrating from the cytoplasm to the plasma membrane where their content was secreted outwards through the zona pellucida, and larger vesicles were observed in the intercellular space. At the blastocyst stage (day E4.5), the secretion of vesicular structures was observed both in the extracellular medium through the zona pellucida, as well as in the blastocoel cavity.

## EVs as mediator and messengers in reproductive biology

**Fig. 2:**
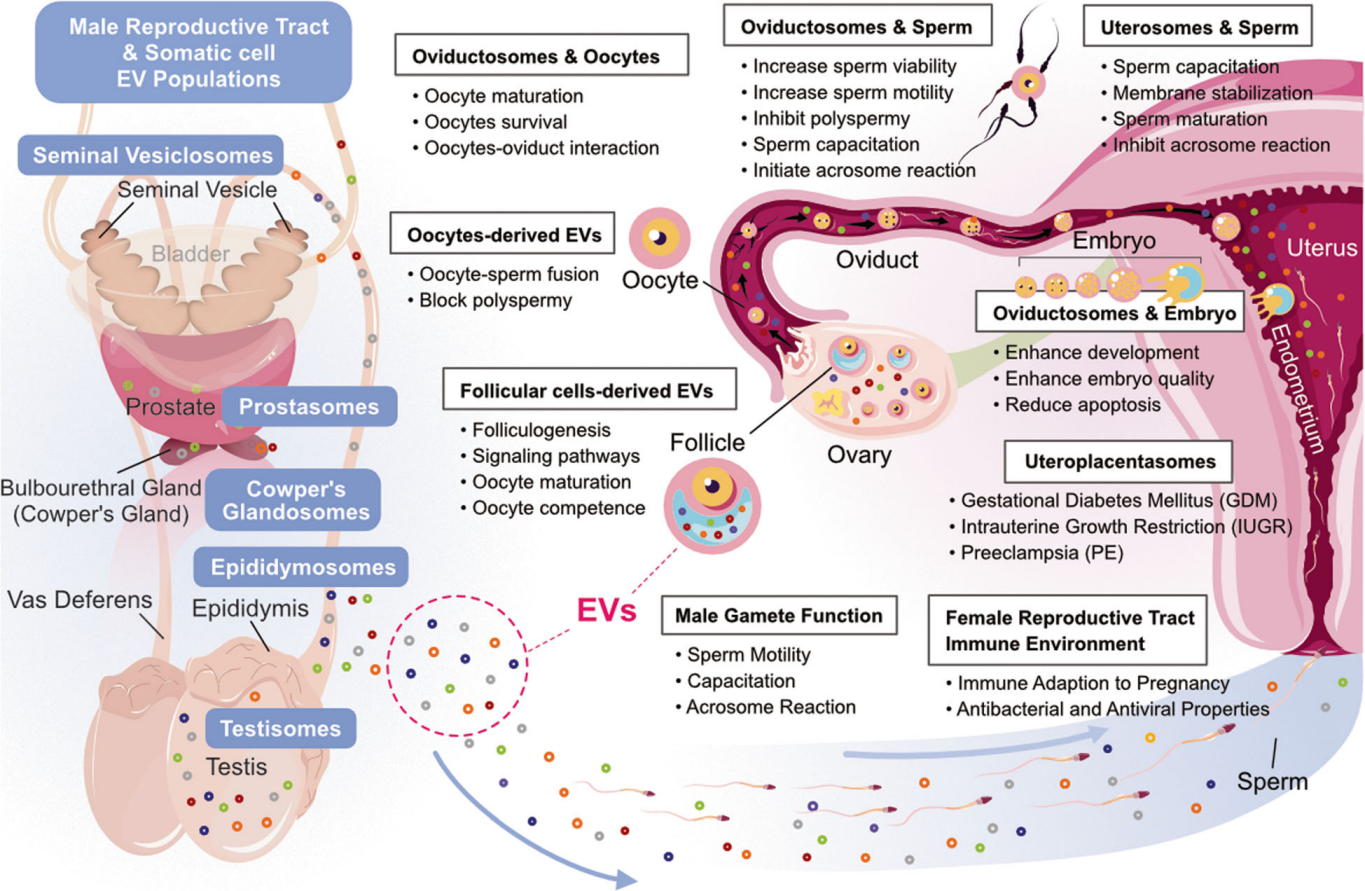
Multifunctional roles of EVs in male and female reproduction organs. Male and female reproductive organ-derived EVs may be involved in sperm and oocyte maturation, sperm-oocyte fusion and also increase embryo viability and pregnancy efficacy

Exosomes play a significant role in the transmission of specific cargo molecules in the reproductive tract to modulate transcription and translational activity, granulosa cell proliferation and differentiation, cumulus expansion, gametogenesis, normal follicular growth, oocyte maturation, fertilization rate, embryo development, blastocyst formation and implantation, pregnancy outcomes, and fertility (Fig. 2). Human reproductive systems are highly dynamic and have well-characterized stages. EVs are involved in the intercellular communication at each stage of the reproductive system in both the male and female reproductive tracts. EVs are associated with reproductive biology and have been identified in different fluids, such as prostatic and epididymal fluid, seminal fluid, follicular fluid, oviductal fluid, cervical mucus, uterine fluid, amniotic fluid, and breast milk, as well as the originating tissues [9, 111–118]. EVs are key regulators of different reproductive processes, such as sperm and ovum maturation, coordination of capacitation/acrosome reaction, prevention of polyspermy, endometrial embryo crosstalk, and embryo development [119]. EVs are released by extravillous trophoblasts (EVTs). The syncytiotrophoblast (STB) is considered to be the main site of EV generation, and these EVs play significant roles in immune modulation, either for innate or adaptive responses [120]. EVs derived from the amniotic fluid are responsible for inflammatory and procoagulant activities [121]. EV-derived breast milk is involved in bone formation, immune modulation, and gene expression regulation, especially for long non-coding RNAs [122, 123]. In vitro and in vivo studies suggest that embryo-derived EVs act as modulators of embryo-to-embryo communication in polytocous species [39, 106]. EVs mediate communication between the inner cell mass and trophectoderm. EVs secreted by bovine embryos can be taken by zona-intact bovine embryos, increase blastocyst rates at d 7 and 8, and improve embryo quality, with significantly decreased apoptotic cells [124]. Conceptus-derived EVs are found in the cytoplasm of luminal epithelial cells and some glandular epithelial cells. These EVs can target the uterine epithelium and serve as a novel form of cell-to-cell communication during the establishment of pregnancy [19]. EVs derived from cervical mucus have sialidase activity, which is involved in modifying highly glycosylated mucus to favor spermatozoa access to the uterine cavity and tubes [113]. Bovine follicular fluid–derived exosomes and cumulus–oocyte complexes from mice and cattle revealed that follicular EVs are taken up by cumulus cells, promoting both cumulus expansion and related expansion of genes [13]. Several studies have shown that exosomes are released from various parts of the female reproductive tract, including the uterus, oviduct epithelium, endometrium, preimplantation embryos, and placental trophoblastic cells [9, 12, 125, 126]. Exosomes play an important role in intercellular communications, which is essential for preconception and post-conception, and also serve as a marker for pregnancy and pregnancy-associated pathologies in humans [127, 128]. Prattichizzo et al. [129] reported that aged-cell-derived exosomes are more proinflammatory than younger cell-derived ones. A mouse study suggested that serum-derived exosomes from young mice were able to mitigate inflammation in both the central and peripheral nervous systems of old mice, which reduces morbidity and mortality caused by age-related diseases [130]. Exosomes contain miRNAs derived from senescent cells that initiate senescence and aging in the surrounding cells. Exosomes contain intracellular miRNAs, such as the let-7, miR-34a, and miR-17-92 cluster, which are involved in regulating and developing mammalian cells [131–134]. Exosomes play a significant role in the removal of waste and biomolecules, which are essential for the maintenance of intracellular proteins, RNA homeostasis, and cellular fitness [135]. During pregnancy, exosomes are derived from various cells and tissues, including placental trophoblasts, embryos, endothelial cells, immune cells, and platelets, which mediate the necessary communication between maternal and fetal circulation during pregnancy [120, 136]. Exosomes derived from the placenta promote endothelial and vascular cell migration, which is an essential step for the establishment of fetal-maternal circulation and remodeling of uterine spiral arteries [137]. Exosomes serve as signaling molecules and are involved in the activation of signaling pathways involved in the regulation of folliculogenesis, oocyte maturation, ovulation, meiotic resumption, embryo development, and fertilization rate [8, 115, 138]. Bioinformatic analysis revealed that 14 and 5 miRNAs were found in follicular fluid of young versus old mares, respectively. Levels of miR-513a-3P, miR-181A, and miR-375 were significantly higher in exosomes, and all these miRNAs suppressed the TGFβ pathway [139]. Exosomes play a significant role in coordinating between the embryo and uterine endometrium, which is required for successful implantation [140, 141]. Extracellular vesicles released from endometrial epithelium contribute to the transfer of miRNA and connective molecules to blastocysts and endometrium, which play a significant role in implantation and fertility outcomes [19, 141]. Exosomes derived from ovine uterine stimulated trophectoderm cells to proliferate and secrete interferon tau via TLR-mediated cell signaling [12]. Placenta-derived microvesicles in the first trimester of pregnancy indicate the role of EVs in maternal-embryo crosstalk during pregnancy [142]. Exosomes released from the fetal membrane during pregnancy potentially transmit signals originating from the fetus to the maternal uterus, as well as the cervix [143].

EVs are stable, versatile, cell-derived nanovesicles with target-homing specificity and the ability to transfer through in vivo biological barriers, and they hold promise for the development of new approaches in drug delivery [23]. EVs are capable of intercellular genetic transfer and can facilitate new diagnostic and therapeutic tools in the field of reproductive biology. EVs serve as potential biomarkers for disorders of reproductive organs. The release of placental EVs is modulated by a number of factors that arise from the placenta, and maternal blood is the source of EVs [144]. Placental–exosomal miRNA cargo is related to cell migration potential and inflammatory cytokine production. Low-oxygen tension exosomes decreased endothelial cell migration potential and increased TNF-α production [145]. Placental EVs play a significant role in infectious diseases during pregnancy. Both total and placental-derived EVs are increased in the plasma of pregnant women with HIV infection compared with non-infected controls [145].

## Effects of extracellular vesicles on placentation and pregnancy disorders

In humans, a successful pregnancy depends on normal placental formation, normal implantation, and development of the placenta, which are responsible for fetal growth and development during pregnancy. Soluble factors are involved in normal placental development through intercellular interactions in various types of cells, including trophoblasts, endothelial cells, immune cells, mesenchymal stem cells (MSCs), and adipocytes [17]. Exosomes are released from decreased insulin sensitivity and glucose uptake in skeletal muscles, contributing to the pathophysiology of gestational diabetes mellitus (GDM) [146]. Secretory levels of exosomes are significantly higher in GDM than those in normal glucose conditions [147]. Exosomes released from other types of cells can affect placental function and are involved in regulating the physiological and pathological mechanisms of pregnancy. For example, exosomes released from adipocytes mediate placental metabolic status and contribute to GDM [147] and are also involved in mediating maternal metabolic changes in pregnancy between different organs and the placenta [147]. Concentrations of circulating placental-derived EVs increase during abnormal placentation in preeclampsia (PE) [148]. During gestational age, the level of exosomes is increased and is involved in regulating the maternal immune response during pregnancy [149–153]. EVs can regulate the expression and production of different cytokines during pregnancy [146, 154]. Placental EVs are believed to play a role in modulating pro-inflammatory and anti-inflammatory states by modulating cytokine release [151]. Placental EVs inhibit maternal immunity and promote fetal survival through the expression of specific immunoregulatory molecules [149–153]. Placental-derived EVs contain syncytin-1, which suppresses the production of tumor necrosis factor alpha (TNF-α) and interferon gamma (IFN- γ), which are inflammatory regulators in early pregnancy [155]. Placental EVs induce the release of proinflammatory cytokines from endothelial cells, including TNF-α, macrophage inflammatory protein (MIP)-1α, interleukin (IL)-1α, -6, -8, and -1β, and activate macrophages to release proinflammatory IL-1β. The activation of phagocytic cells regulates the maternal immune response to maintain a normal pregnancy and protects against infection [156, 157]. Circulating EVs induce the formation of pro-inflammatory environments and endothelial cell dysfunction in the placenta, and contribute to the formation of pro-inflammatory environments and endothelial cell dysfunction in the placenta [18]. Circulating EVs also facilitate the prediction of the physiological and pathological conditions of the cell of origin. EVs derived from trophoblasts increase the migration of monocytes through the production of IL1B, IL6, SERPINE1, and colony stimulating factor 2 (CSF2) [157]. EVs derived from PE patients inhibit the proliferation of macrophages and the expression of inflammatory cytokines, such as IL-12 and TNF [158]. Maternal plasma-derived EVs contain miR-548c-5p, which causes inflammatory responses and PB in pregnant mice [159]. A mouse study revealed that EVs derived from injured placenta induce PE-like symptoms, such as hypertension and proteinuria, by inducing endothelial injury, vasoconstriction, and hypercoagulation [160]. The secretary level of exosomes are significantly are higher in compared to normal glucose condition [161].

### Role of extracellular vesicles in normal pregnancy and pregnancy-related diseases

**Fig. 3.**
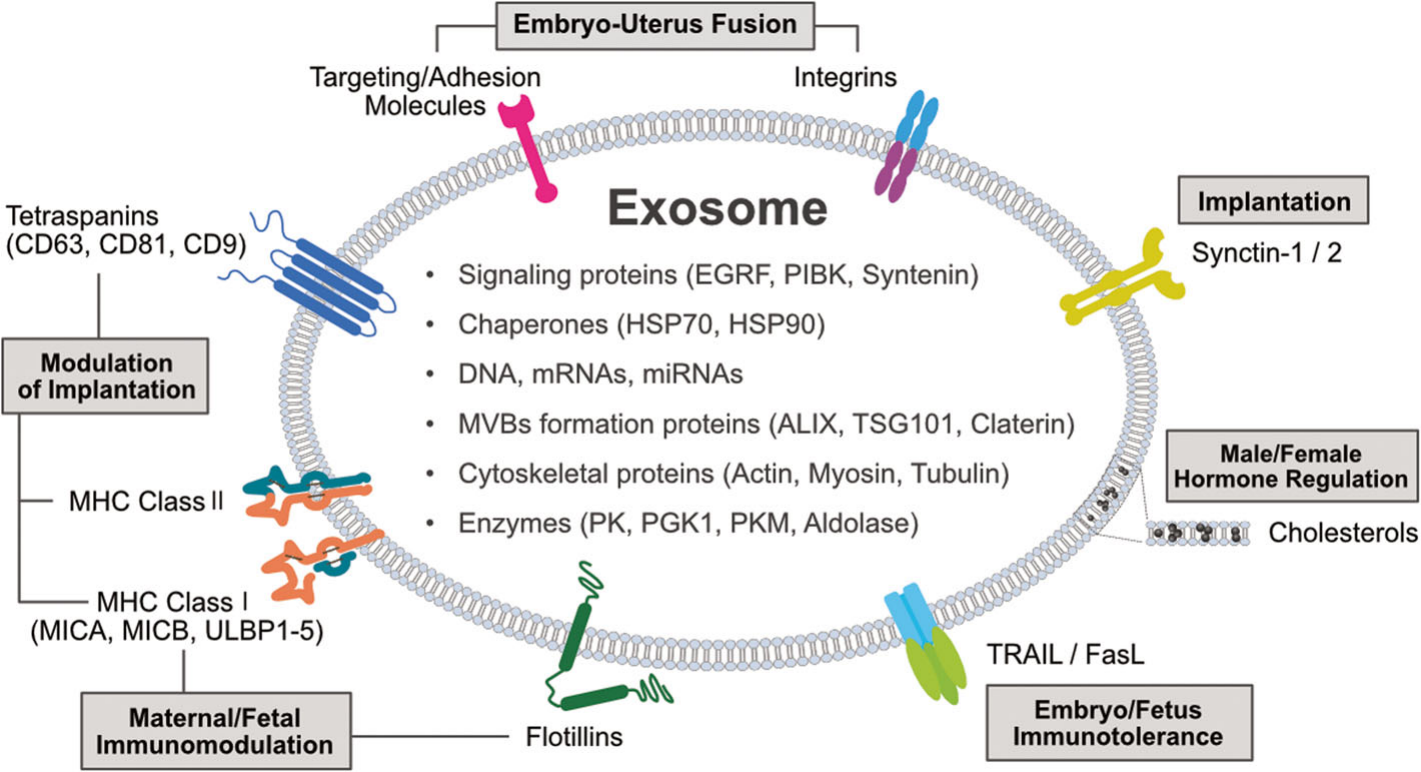
Typical structure of EVs, properties, and functional attribution of EVs on female reproductive system. EVs are involved in various physiological functions including embryo-uterus fusion, modulation of implantation, immunomodulation, regulation of male and female hormone regulation, immunotolerance of embryo

EVs are playing an important role in intercellular communication through the transfer of a wide spectrum of bioactive molecules, contributing to the regulation of diverse physiological and pathological processes, and mediating fetal–maternal communication across gestation. EVs play a significant role in maternal-embryo interaction within the human uterine microenvironment, promoting implantation, the earliest and essential step for successful pregnancy. Studies have suggested that exosomes can be transferred between the fetus and maternal bodies [159]. EVs potentially regulate multiple processes of pregnancy, such as implantation, migration, and invasion of trophoblasts, and cellular adaptations to physiological changes (Fig. 3) [120, 162, 163]. Alterations in EVs are critically involved in pregnancy-related diseases. Moreover, EVs have shown great potential as biomarkers for the diagnosis of pregnancy-related diseases. The concentration, composition, and bioactivity of EVs can regulate pregnancy-related diseases [144, 164]. EVs are playing significant role in maternal-embryo interaction within human uterine microenvironment, promoting implantation, an earliest and essential step for successful pregnancy [161, 165]. Exosomes from endometrial epithelial cells (ECs) were treated with estrogen, or estrogen, and progesterone (EP); EP-treated ECs have exosomes that contain proteins associated with embryo implantation and extracellular matrix remodeling [26]. MicroRNAs (miRNAs) play a potential role as mediators of embryo-endometrium crosstalk in the implantation process [166, 167]. The interaction between EVs and immune cells modulates pregnancy, tolerates the growing fetus, and maintains its normal functions [168, 169]. EV-derived heat shock protein family E member 1 (HSPE1) promotes Treg differentiation from CD4^+^ T cells and Treg cell expansion [170]. Exosomes derived from macrophage-derived exosomes increase the release of pro-inflammatory cytokines, such as IL-6, IL-8, and IL-10, potentially facilitating protective placental immune responses during pregnancy [171]. Exosomes are derived from primary human placental trophoblasts containing chromosome 19 miRNA cluster (C19MC) miRNAs that attenuate viral replication in recipient non-placental cells by upregulating autophagy [172]. EVs are involved in metabolic homeostasis and are associated with metabolic regulation during pregnancy. Placental-derived exosomes are able to increase insulin-induced glucose uptake in the skeletal muscle of diabetic patients, suggesting that placental exosomes may engage in changes in insulin sensitivity in normal pregnancies [146]. Exosomes from adipose tissue (AT) of pregnant women with normal glucose tolerance affect the expression of glucose metabolism-related genes in placental cells [173]. Exosomes from extravillous trophoblast cells cultured under low oxygen tension increased TNFα expression in HUVECs [174]. Exosomal miR-141 derived from fetal trophoblasts induces T cell proliferation, indicating that placental EVs regulate maternal immune cells and cause immune disorders during pregnancy [175–177]. Exosomes from GDM pregnancies increase the release of pro-inflammatory cytokines from ECs, including GM-CSF, IL-6, and IL-8 [161]. Preterm labor (PTB)-enriched exosomes are associated with inflammatory molecules that affect the labor process [178].

### Role of extracellular vesicles in mother and embryo cross-communication

EVs play a significant role in paracrine communication between the mother and embryo [105] and are involved in synaptic plasticity, deliver neurotransmitter receptors, and modulate tissue regeneration [179]. EVs participate in regulating immune responses, particularly triggering the adaptive immune response and suppressing inflammation [180]. A previous study reported that EVs play important roles from preconception, gamete maturation to implantation and throughout pregnancy [141]. Human uterine fluid-derived EVs contain a specific subset of miRNAs that are not detectable in maternal cells by the human endometrial epithelial cell line ECC1 [11]. Similarly, the uterine fluid of pregnant sheep contains EVs positive for CD63 and HSP70, as well as small RNAs and miRNAs [181]. Exosomes derived from human endometrial epithelial cells are subject to steroid hormonal regulation by estrogen and progesterone and vary with the menstrual cycle [26]. Internalization of miR30d by mouse embryos via the trophectoderm increased the overexpression of adhesion-related genes, *Itgb3*, *Itga7*, and *Cdh5*, and also increased embryo adhesion; conversely, miR-30d deficiency results in reduced implantation rates and impaired fetal growth [182]. Heterogeneous nuclear ribonucleoprotein C1 (hnRNPC1) is involved in cell-to-cell communication, and previous studies suggest that maternal endometrial miRNAs act as transcriptomic modifiers of the preimplantation embryo [183, 184]. Exosomes released from human endometrial epithelium transferring molecular cargoes promote implantation to the blastocyst and endometrium [11]. A bovine model study demonstrated that embryo-derived EVs improved the growth and viability of cloned bovine embryos and increased implantation rates and full-term calving rates [185]. Mouse studies suggest that microinjections of 3-5 days old blastocysts with embryonic stem (ES) cell-derived EVs before transfer into surrogate mothers significantly increased the likelihood of implantation. In addition, ES cell-derived EVs improve the capability of TE cells within the blastocyst to migrate into the uterus and promote blastocyst implantation [20]. EVs from day 17 of pregnancy induce apoptosis of immune cells and primary endometrial epithelial cells (EECs) through increased expression of apoptosis-related genes, including *BAX*, *CASP3*, and *TNFA*, which are required for conceptus implantation, during which a portion of the endometrial epithelium disappears [186].

### Role of microRNAs in embryo implantation

miRNAs are small non-coding RNAs that function in RNA silencing and post-transcriptional regulation of gene expression. They regulate genes expression in blood plasma and serum, as well as other body fluids, and are involved in intercellular communication [187–189]. miRNAs are secreted by all types of cells, and the concentration of extracellular miRNAs is associated with physiological and pathological conditions of the body [190, 191]. Embryo implantation is a crucial step in the establishment of pregnancy in mammals and has a profound effect in reproductive efficiency. The process of implantation is under the strict regulation of ovarian hormones, estrogen, and progesterone [192]. Several molecules, such as cytokines, chemokines, growth factors, lipids, and receptors also participate in the regulation of implantation through autocrine, paracrine, and juxtacrine pathways [192]. Several studies have reported that miRNAs are involved in the regulation of oogenesis, fertilization, implantation, and placentation. Dysregulation of miRNAs causes reproductive disorders, such as polycystic ovarian syndrome and endometriosis [193, 194]. miRNAs are associated with various types of proteins such as the AGO family, nucleophosmin 1, bound to lipoproteins and apoptotic bodies [73, 195, 196]. These enzymes are involved in miRNA biosynthesis pathways, such as DICER; AGO2 leads to embryonic death around gastrulation, suggesting an important role of miRNAs in early embryonic development [197–199]. Regulation of the expression of Dnmt3a/b by miR-29b causes disruption of DNA methylation, which leads to early embryonic developmental blockade in mice [200]. Inhibition of this miRNA significantly reduces morula and blastocyst formation [201]. Liu et al. reported that 45 miRNAs were differentially expressed between dormant and activated mouse embryos; particularly, let-7a levels were highly expressed in dormant embryos and inhibited the expression of Dicer and prevented embryo implantation [202, 203]. A porcine embryo study suggested that there was a lower expression of miR-24 in the blastocyst stage than in in vitro fertilized (IVF) embryos [204]. Another study suggested that high level expression of miR-24 inhibited the development of embryos to the blastocyst stage [36]. Differential expression of miRNAs was observed between IVF bovine blastocysts and degenerate embryos, and relatively higher levels of miR-181a2, miR-196a2, miR-302c, and miR-25 were found in degenerate embryos [35]. Variable expression patterns of miRNAs were observed in human endometrial fluid secreted by the endometrial glands at different stages of the menstrual cycle [182]. Placenta-specific miRNAs, such as miR-515-3p, miR-517a, miR-517c, miR-518b, miR-526b, and miR-323-3p, are widely expressed in the blood plasma of pregnant women [205, 206]. A cow model study demonstrated that circulating EV-derived miRNA is not only able to identify pregnancy, but can also distinguish between successful implantation and embryonic mortality at the early stage of pregnancy [207]. EVs derived from bovine follicular fluid contain miRNAs that reflect the stage of the estrus cycle and can modulate cumulus cell transcription during in vitro maturation [208]. Murine oviductal tract EVs (oEVs) contain miR-34c-5p, which is transferred to the sperm heads, promoting the first cleavage in the zygote and controlling embryonic development [209]. EVs secreted by donor oviductal cells increase birth rates after embryo transfer in mice due to decreased apoptosis and improved cellular differentiation in embryos [210].

### Role of EVs in animal reproduction

Cell communication is a crucial process for several molecular processes involved in female reproduction. EVs have been identified as one of the key players in regulating temporal sequences, spatial interaction, and cell-cell signaling in all events in sexual reproduction [211]. EVs have been observed in seminal fluids to modulate sperm capacitation in humans [212] and pigs [213] and also influence female physiology by modulating immune-related gene expression in the porcine endometrium [214]. EVs from avian uterine fluid may play an essential role in preserving sperm function [215]. EV-mediated molecules are produced by somatic cells and germ cells present in follicular fluids (FF). EVs derived from FF have been used in various animal models, such as horses [8], humans [216] and cows [217]. Follicular fluid comprises a heterogeneous EV population secreted by granulosa, cumulus, and somatic follicular cells with functions related to the control of steroidogenesis [8, 216, 217]. EVs play a significant role in reproductive processes as intercellular communicators and are found in follicular fluid [217], oviductal fluid [9] and secreted by embryos in culture media [35–37]. Intercellular communication within the microenvironment of the ovary is essential for oocyte and follicle development. EVs are secreted by follicular cells and are found in follicular fluid, which transmit information between cells [217]. Sohel et al. [125] reported that the majority of miRNAs from follicular fluid were in the exosome fraction, and exosome uptake by follicular cells was associated with an increase in miRNA levels in these cells [125]. Exosomes derived from follicular fluid regulate TGF-β signaling pathways; they have been shown to regulate ACVR1 and ID2 in granulosa cells in vitro by transferring mRNA, protein, and miRNAs in follicular development of granulosa cells [218]. EVs are a component of the oviductal fluid that favors oocyte and embryo quality [14]. Proteomic analysis revealed that EVs secreted proteins, such as oviductal glycoprotein (OVGP), heat shock protein A8 (HSPA8), and myosin 9 (MYH9) by bovine oviduct epithelial cells (BOECs), which are involved in fertilization, early pregnancy development, and zona pellucida maturation [219, 220]. Embryos treated with EVs from BOEC culture media induced an increased number of total cells and better survival rate after vitrification compared to embryos cultured without EVs [14]. Oviductal fluid-derived EVs from the isthmus resulted in the greatest bovine embryo survival rate after vitrification. *AQP3* (Aquaporin 3) was upregulated in embryos supplemented with EVs from the isthmus compared to embryos supplemented with FCS only [221]. Supplementation of exosomes secreted by somatic cell nuclear transfer (SCNT) embryos in the culture medium of SCNT embryos increased blastocyst rate, total cell numbers, ratio of ICM/TE, and transcript levels of *OCT-4* in comparison to SCNT embryos without supplementation [185]. Exosomes present in the culture medium are essential for embryo development, and changes during embryo development caused by culture medium replacement may be repaired by exosome supplementation [185]. Progesterone treatment increased the number of EVs in the uterine lumen compared to that in the P4 receptor antagonist group [222]. EVs derived from porcine trophectoderm induce aortic endothelial cell proliferation, which may stimulate angiogenesis [223]. Furthermore, porcine trophectoderm and aortic endothelial cell EVs have miRNAs predicted to modulate angiogenesis and placental development pathways, suggesting that these EVs may play an important role in the communication between the conceptus and maternal endometrium, influencing the establishment of pregnancy [223]. Small extracellular vesicles (sEVs) released from endometrial epithelial cells (EECs) activate signaling pathways in trophoblasts, thus promoting migration and invasion, which affect implantation rates. These sEVs serve as novel intercellular communication mechanisms during embryo implantation [224].

The success of pregnancy depends on the molecular dialogue between the embryo and the female reproductive tract that starts at the oviduct and continues until the placenta is formed. Cytokines and growth factors, such as interleukin-1β (IL-1β), heparin-binding epidermal growth factor (HB-EGF), integrins, and leukemia inhibitory factor (LIF), act synergistically in embryo-maternal crosstalk. For example, the expression of epithelial cell adhesion proteins increases endometrial receptivity by IL-1 [225] and stimulates angiogenesis to promote embryonic growth [226]. HB-EGF receptors on the surface of the embryo and endometrium facilitate implantation and promote the development of blastocyst [227, 228].

### Roles of extracellular vesicles in the male reproductive system

**Fig. 4:**
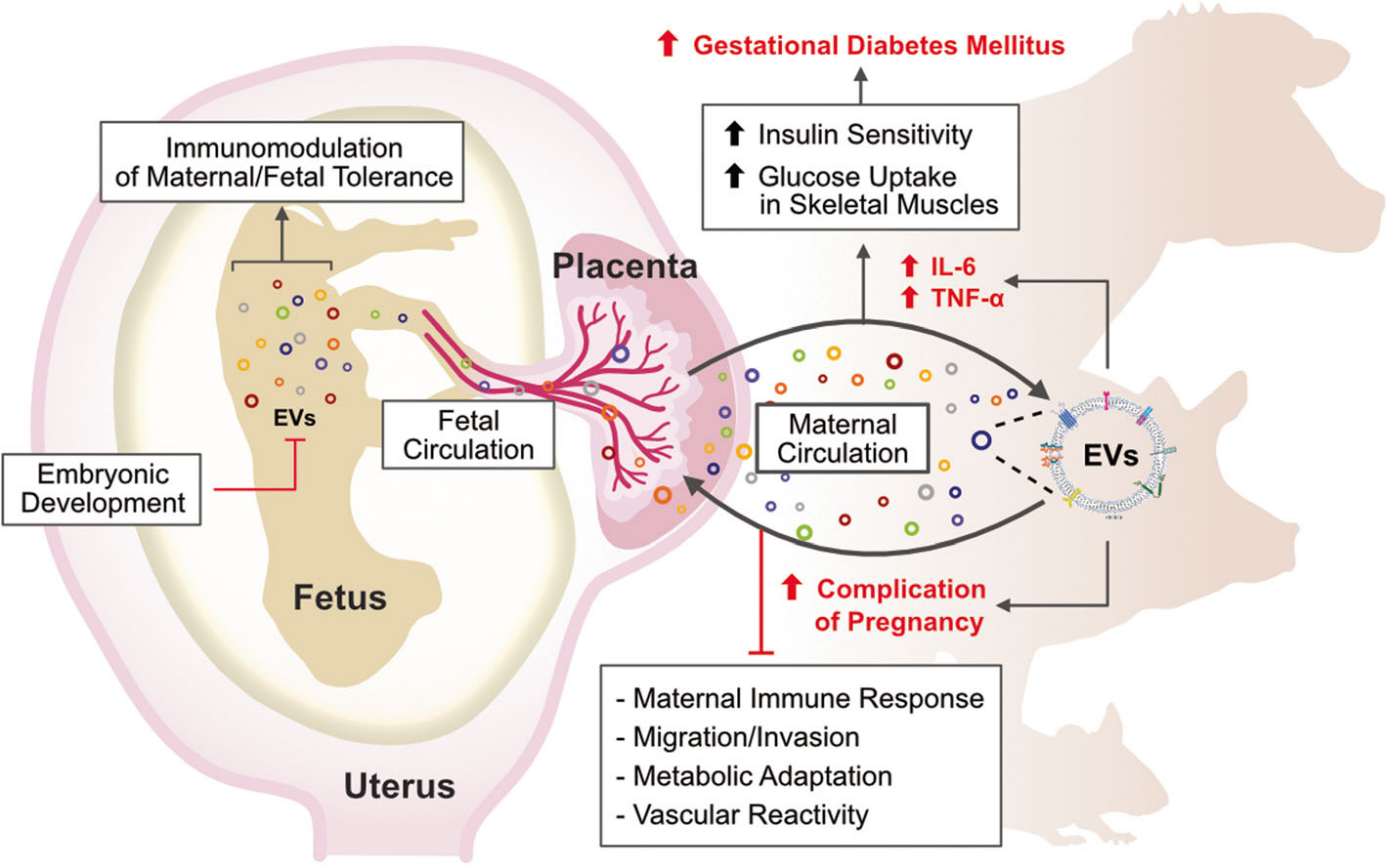
Role of EVs in normal pregnancy and pregnancy related diseases. EVs mediate fetal-maternal communications in normal pregnancy. EVs contribute to embryo implantation by promoting trophoblast adhesion. Placenta can interact with immune cells via EVs to balance immune activation and suppression across the gestation. EVs are involved in angiogenesis, immunomodulation, glucose metabolism, embryonic development and fetal circulation. Particularly, secreted exosomes play a critical role in cell-cell communication mediators in pathological scenarios. As a result, these can induce gestational diabetes

EVs are important regulators of the biological function of sperm and seminal fluid in normal and pathological reproduction [105]. EVs of the male reproductive tract are a product of a diverse population of cells, and are conserved, abundant, and carry a complex payload of regulatory elements that support sperm function, which is essential for effective functions of female reproductive tract biology after mating (Fig. 4) [229, 230]. Epididymosomes are a heterogeneous population of EVs that are produced by epithelial cells lining the epididymis and have an average size between 50 and 250 nm [231, 232]. They play a significant role in mediating post-testicular sperm maturation and storage across mammalian species. They can tether and transiently fuse with sperm to facilitate protein transfer, and are also involved in sperm maturation and successful fertilization [233–235]. Epididymosomes contain sncRNA cargo and are directly implicated in the transfer of microRNAs (miRNAs) and transfer RNA-derived RNA fragments (tRFs) to epididymal sperm [236–239]. Epididymosomes of mice, humans, and bulls contain an abundance of antioxidant enzymes, which play an important role in the elimination of defective sperm in humans, rats, and cattle [240–242]. Epididymosomes also play a significant role in sperm maturation and storage, and cargoes of epididymosomes have been shown to influence the female reproductive tract [243, 244]. Seminal fluid EVs (SFEVs) isolated from vasectomized men lack epididymosomes and show reduced capacity to support motility, capacitation, and initiation of the acrosome reaction, compared to the SFEV pool of intact men [245]. The interactions between epididymosomes and sperm are potentially involved in the female reproductive tract, which assists sperm in attaining full functional maturity [245].

SFEV is not only involved in modulating sperm function, but also has an impact on the immune environment within the female reproductive tract [230]. The interaction between seminal fluid EVs and the female reproductive tract of epithelial cells initiates an inflammatory response, similar to the responses observed following exposure to seminal fluid [214, 246–249]. EVs of the reproductive tract are potentially involved as fundamental regulators of reproductive success by regulating male gamete function and influencing the female reproductive tract during pregnancy. However, alterations in EV composition not only regulate impaired fertility, but could also influence fetal development and impart long-term consequences for offspring health.

Human seminal plasma is a complex fluid produced by secretions from several glands of the male genital tract and male gametes consist of rich amount of EVs [250]. EVs are synthesized by the prostate, as well as by the epididymis, and even by the testis. EVs are classified based on testicular, prostatic or epididymal localization. EVs from seminal plasma involved in various aspects of male fertility, improving sperm function by regulating the timing of sperm capacitation, inducing acrosome reaction, stimulating sperm motility enabling them to reach the ovocyte [251]. Myelinosomes are secreted by Sertoli cells and secretory organelles loaded with specific cargoes and capable of leaving the cell in their entirety, in the form of extracellular vesicles [252]. Prostasomes are nanosized exosomes secreted by the acinar lumen of prostate epithelial cells. These prostasomes contain proteins, lipids and nucleic acids. Prostasomes contains rich level of cholesterol and sphingomyelin, with a particularly high cholesterol/phospholipid ratio [253]. Prostasomes play significant role in controlling capacitation and the acrosome reaction and also involved in preventing premature capacitation of spermatozoa and premature acrosome reaction. The prostasomal membrane is enriched in cholesterol, which contributes to its stability in the acidic vaginal environment [212]. Bovine model studies demonstrated that epididymosomes contain several proteins that are involved in the acquisition of sperm mobility, fertilisation capacity and protection against oxidative stress. The content of these vesicles also depends on the region of the [234, 254] epididymis. EVs are secreted by testis which contains sperm RNA is an important epigenetic player in the early development of the embryo and the health of the offspring [255]. Altogether, these findings suggest that the heterogeneous population of EVs present in seminal plasma is known to influence sperm functions [141, 256].

### Role of EVs in reproductive and therapeutic medicine

EVs play an important role in both physiological and pathological processes as biomarkers of fertility, reproductive cancer, embryo quality, placenta quality, and early abortion [211]. The circulating level of EVs depends on the physiological level of tissues, serum or other biological fluids, animal model, time, and type of disease. In particular, EVs regulate a variety of physiological processes. Cargoes present in EVs are protected from degradation and can be used as biomarkers for non-invasive cancer diagnosis in various types of reproductive cancers [188, 257, 258]. Physiological and pathological conditions influence EV concentration, cargo, and function. Several miRNAs have been established as biomarkers in the ovarian follicle, which create a suitable microenvironment for the growth, maturation, and fertilization of oocytes [259, 260]. For example, expression of miRNA-375 in granulosa cells and oocytes facilitates follicular growth proliferation, spread, and apoptosis of cumulus cells, whereas overexpression of miR-375 inhibits the ability to proliferate, increases the apoptosis rate of cumulus cells in cows, and suppresses estradiol production and follicular development in porcine granulosa cells [261–264]. Placenta-derived EVs can induce differentiation due to the presence of placenta-specific proteins (e.g., PLAP4) and miRNAs (e.g., chromosome 19 miRNA cluster) that are exclusively expressed in the placenta and serve as biomarkers of maternal-fetal health and evolution and for diagnosis of preeclampsia [265–267]. EVs show immense therapeutic potential in various diseases, including reproductive cancers, such as ovarian cancer. For example, amniotic derived EVs have been used to treat endometritis in mare to attain successful pregnancy [268]. Zhang et al. [269] demonstrated that transplantation of menstrual blood-derived stromal cells (MenSCs) derived sEVs safely and effectively promoted the regeneration of endometrial glands and blood vessels, and improved fertility in IUA rats. Furthermore, treatment with MenSCs and MenSCs-sEVs increased BMP7 levels and activated the SMAD1/5/8 and ERK1/2 pathways in vivo, thereby alleviating endometrial fibrosis by inhibiting TGFβ1/SMAD3 signaling.

### EVs are serving as signaling molecules to target various types of reproductive cells

EVs are serving as intercellular signaling molecules are considered imperative for the regulation and accomplishment of different physiological events including cellular proliferation and differentiation, gametogenesis, fertilization, and embryonic development [141]. The success of pregnancy greatly depends on gametogenesis, fertilization, and an adequate uterine environment for embryonic development [270]. These highly complex processes greatly rely on the crosstalk between the gametes and the different segments of the reproductive tract. EVs are regulating diverse signaling pathways in targeting the cells [271]. EVs secreted by the male reproductive tract including epididymosomes and prostasomes are significant role in the maturation process of sperm [272, 273]. EVs derived from the uterine fluids of murine exhibited the expression of certain sperm essential proteins including spermadhesionmolecule 1 (SPAM1) and plasma membrane calcium pump (PMCA4) [116, 274]. Oviductosomes contains cargoes including proteins aV integrin, CD9 tetraspanin, heat shock proteins, lactadherin oviductal specific glycoprotein (OVGP), lipids, SPAM1, RNAs, and miRNAs are involved in acrosome reaction, increases sperm viability and motility, reduces the incidence of polyspermy through zona hardening, induces the phosphorylation of sperm-associated proteins during capacitation, and modulates fertilization, fertilization and early embryo development [275–281]. Various type of factors including insulin, transforming growth factor-beta (TGFB) and wingless/Int (WNT) signaling members [282, 283] growth factors [284] and hormones [285] are involved in both the folliculogenesis and initiation of different signaling pathways. Tetraspanins including CD9 and CD81 involved mediating the oocyte-sperm fusion process and exclusively CD81 may facilitate the transfer of CD9 from the oocytes to the sperm plasma membrane [286]. Oviduct-derived EVs are involved in embryonic development through mediating the embryo–maternal interactions during early embryonic development, leading to improved embryo quality and successful pregnancy [287]. Endometrial-epithelial derived EVs not only facilitate endometrium-embryo crosstalk but also help in the implantation of the embryo. EVs are derived from trophoblasts carry molecules such as miRNA and significant proteins are involved in the normal placental function and angiogenesis within the trophectoderm. Further, the trophectoderm derived EVs penetrate and stimulate the proliferation of maternal endothelial cells [157]. High concentrations of p38 MAPK in EVs influencing parturition and are involved in in inflammatory responses, cell proliferation, apoptosis, and stress induced signaling [288]. All these findings suggest that all the functional molecules carried by the EVs potentially modulate different reproductive events such as gametes maturation, fertilization, and blockage of polyspermy, development, and implantation of the embryo, fetal development, and parturition.

## Conclusion and future perspectives

**Fig. 5:**
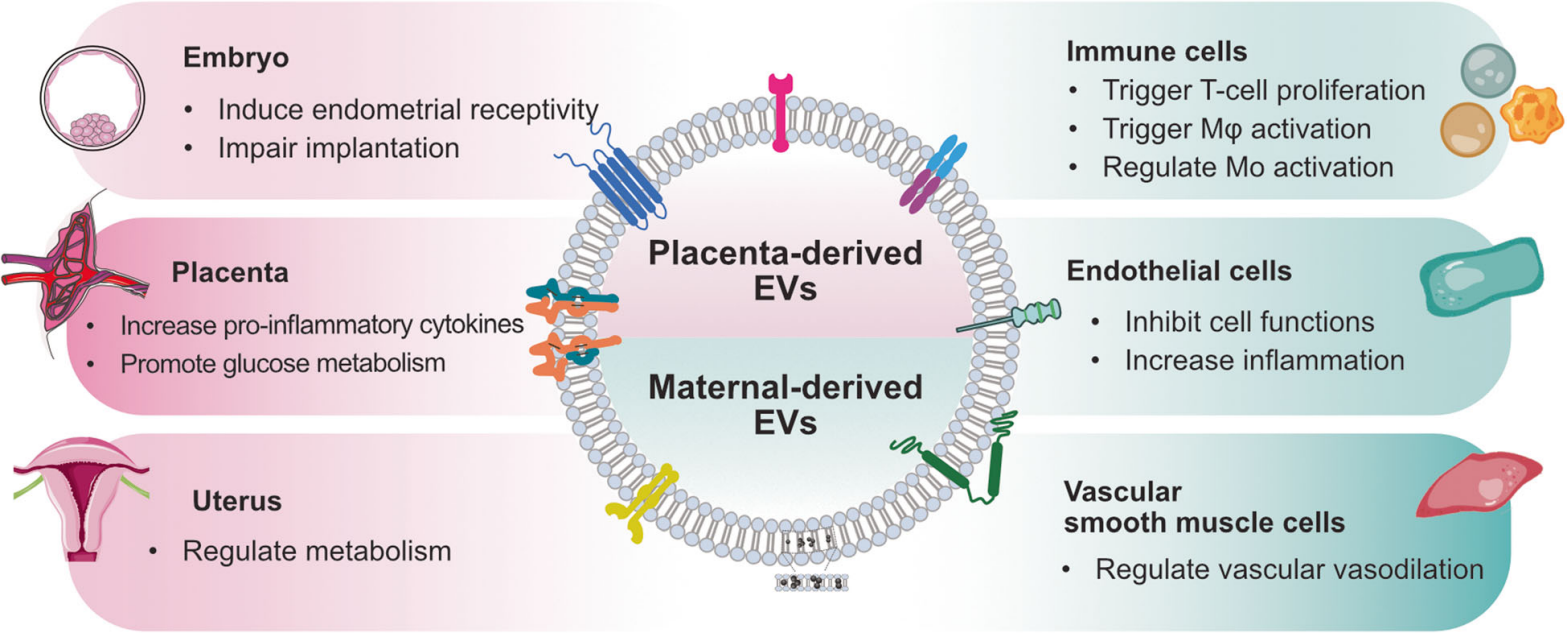
Effects of EVs in pregnancy-related diseases. Concentration and composition of EVs are released from male and female reproductive systems are involved in pregnancy-related diseases. EVs mediate dysregulation of the balance between pro- and anti-inflammatory responses in immune cells and placenta. EVs are play significant role in induction of endometrial receptivity, uterus regulation, increases pro-inflammatory cytokines, increases glucose metabolism and regulation of vascular cells

Extracellular vesicles (EVs) are a heterogeneous population of cellular couriers and membraned structures secreted by cells that contain various biomolecules, such as proteins, lipids, RNAs, and DNAs, which can serve as long distance messengers and play a significant role in cellular communication and cell function. EVs differ in size and function. EVs have various subsets, including exosomes, microvesicles, and apoptotic bodies. These vesicles play a significant role as key regulators of human reproduction. EVs are located in various body fluids that are critical for reproduction, such as follicular fluid, endometrial fluid, semen, and Fallopian tubal fluid [105, 141]. EVs can carry significant phenotype-altering cargo, such as transcription factors and microRNAs. EVs can serve as excellent carriers for drug delivery because of their ability to transfer their contents to target cells. Several studies have documented that EVs carrying functional molecules control different reproductive events, such as gamete maturation, fertilization, blockage of polyspermy, embryo development and implantation, fetal development, and parturition. The concentration of EVs determines the physiological or pathological state of different reproductive events and may be used as markers for pregnancy term, fetal growth, placental function, and diagnosis of different pathological conditions. EV-mediated cellular communication facilitates the enhancement of diagnostics and therapeutics for fertility-related issues, pregnancy-associated abnormalities, and pregnancy loss. EV communication may provide a foundation for a better understanding of the conception and implantation processes. EVs are released from the placenta into the maternal circulation and have a wide range of functions to regulate immunologic responses to pregnancy and to establish maternal vascular function (Fig. 5). Placenta-derived EVs contain various miRNAs and proteins that play a role in the maintenance of pregnancy in the trophoblast and placental microenvironment. EVs may be utilized as disease biomarkers and drug delivery systems, which provide the opportunity for diagnostic potential with reduced invasiveness in a targeted manner. EVs play important roles in regulating cellular functions and contributing to pregnancy-related diseases. The potential diagnostic value of EVs in pregnancy depends on the concentration and content of circulating EVs. EVs support male gamete function and interact with female reproductive tract cells, are eventually involved in pregnancy, and are potentially involved as regulators of male reproductive success.

To improve the physical and molecular characterization, action, and functions of EVs, there is a need to develop more reliable isolation methods and more sensitive technologies. Although data have demonstrated the potential role of EVs in reproductive physiology and pathology, further investigations are required in this area. Furthermore, it is necessary to understand the molecular mechanism by which EVs regulate key events in pregnancy, which may help elucidate how maternal-fetal communication is established in both normal and pathologic conditions. Detailed studies are required to understand the physiological activity of EVs during early pregnancy, which could open a new avenue to overcome abnormal placentation and pregnancy disorders as well as to characterize differences in the cargo of EVs between pregnant and non-pregnant or embryonic-mortality animals, which ultimately improves fertility rates in agriculturally important animals. In order to improve fertility, it is important to increase our knowledge regarding the biology of EVs in reproductive tissues, which can create a better environment to produce embryos in vitro and consequently generate healthier pregnancies in animals and humans. Although there is considerable evidence that EVs serve as natural therapeutic agents that are able to maintain reproductive success, the progress of reproductive and obstetric-related disorders is still in its infancy, and further investigations that utilize homogeneous and human-specific material are needed. Hence, comprehensive studies on the molecular mechanisms and functional roles of EVs in both male and female reproductive systems are required to decipher the relationship between EVs from various tissues and the entire reproductive process. More studies are required to provide insight into the functions of EVs in pregnancy and to apply EVs to the diagnosis, monitoring, and treatment of pregnancy-related diseases. These studies would provide significant knowledge regarding reproductive mechanisms and contribute to the development of new therapeutic strategies to treat various reproduction-related diseases. It is necessary to address the knowledge gaps in male gamete quality and the composition of seminal plasma on pregnancy outcomes and offspring health.

## Data Availability

Not applicable.

## Abbreviations

AFM: Atomic force microscopy
Alix: ALG-2-interacting protein X
AQP3: Aquaporin 3
AT: Adipose tissue
BAX: BCL2 associated X protein
BF: Blastocoel fluid
BMP7: Bone morphogenetic protein 7
BOECs: Bovine oviduct epithelial cells
C19MC: Chromosome 19 miRNA cluster
CASP3: Caspase 3
CD4: Cluster of differentiation 4
CD63: Cluster of differentiation 63
CD81: Cluster of differentiation 81
CD9: Cluster of differentiation 9
Cdh5: Cadherin 5
CX3CL1/fractalkine: C-X3-C motif chemokine ligand 1
CXCL12: C-X-C motif chemokine ligand 12
Dnmt3a: DNA methyltransferase 3 alpha
Dnmt3b: DNA methyltransferase 3 beta
EECs: Endometrial epithelial cells
EP: Estrogen and progesterone
EpCAM: Epithelial cell adhesion molecule
ESCRTs: Endosomal sorting complexes required for transport
ER: Endoplasmic reticulum
ERK: Extracellular signal-regulated kinase
EVs: Extracellular vesicles
EVTs: Extravillous trophoblasts
FWHM: Full width at half maximum
GDM: Gestational diabetes mellitus
GM-CSF: Granulocyte-macrophage colony-stimulating factor
GTPase: Rab guanosine triphosphatase
HER2: Human epidermal growth factor receptor
HnRNPC1: Heterogeneous nuclear ribonucleoprotein C1
HB-EGF: Heparin-binding epidermal growth factor
HSP70: 70 kilodalton heat shock proteins
HSPA8: Heat shock protein A8
HSPE1: EV-derived heat shock protein family E member 1
ICAM3: Intercellular adhesion molecule 3
ICM: Inner cell mass
IFN-γ: Interferon gamma
IL: Interleukin
ILVs: Intraluminal vesicles degradation of their contents
Itgb3: Integrin subunit beta 3
Itga7: Integrin subunit alpha 7
LIF: Leukemia inhibitory factor
MAPK: Mitogen-activated protein kinase
miRNAs: MicroRNAs
MLCK: Myosin-light chain kinase
MVB: Microvesciles
NTA: Nanoparticle tracking analysis
oEVs: Oviductal tract EVs
OVGP: Oviductal glycoprotein
PBS: Phosphate-buffered saline
PE: Preeclampsia
PLAP4: Placenta-specific proteins
PSA: Prostate-specific antigen
PS: Phosphatidylserine
ROCK1: Rho-associated protein kinase
SERPINE1: Serpin family E member 1
TSG101: Tumor susceptibility 101
VDAC1: Voltage dependent anion channel 1
MenSCs: Menstrual blood-derived stromal cells
MHC: Major histocompality
MIP-1α: Macrophage inflammatory protein
MYH9: Myosin 9
PTB: Preterm labor
SC: Stem cells
SCNT: Somatic cell nuclear transfer
SEM: Scanning electron microscopy
SFEVs: Seminal fluid EVs
SMAD: Mothers against decapentaplegic homolog
STB: Syncytiotrophoblast
TNFA: TNF alpha
TNF- α: Tumor necrosis factor alpha
